# The Effect of Long-Term Administration of Fatty Acid Amide Hydrolase Inhibitor URB597 on Oxidative Metabolism in the Heart of Rats with Primary and Secondary Hypertension

**DOI:** 10.3390/molecules23092350

**Published:** 2018-09-14

**Authors:** Michał Biernacki, Wojciech Łuczaj, Iwona Jarocka-Karpowicz, Ewa Ambrożewicz, Marek Toczek, Elżbieta Skrzydlewska

**Affiliations:** 1 Department of Analytical Chemistry, Medical University of Bialystok, Mickiewicza 2D, 15-222 Bialystok, Poland; michal.biernacki@umb.edu.pl (M.B.); wojciech.luczaj@umb.edu.pl (W.Ł.); iwona.jarocka-karpowicz@umb.edu.pl (I.J.-K.); ewa.ambrozewicz@umb.edu.pl (E.A.); 2 Department of Experimental Physiology and Pathophysiology, Medical University of Bialystok, Mickiewicza 2A, 15-222 Bialystok, Poland; marektoczek@interia.eu

**Keywords:** heart, URB597, hypertension, oxidative stress

## Abstract

Fatty acid amide hydrolase (FAAH) inhibitor [3-(3-carbamoylphenyl)phenyl] *N*-cyclohexylcarbamate (URB597) may influence redox balance and blood pressure through the modulation of endocannabinoids levels. Therefore, this study aimed to compare changes in oxidative metabolism and apoptosis in the hearts of rats with spontaneous hypertension (SHR) and secondary hypertension (11-deoxycorticosterone acetate; DOCA-salt rats) treated by URB597 via intraperitoneal injection for 14 days. The results showed that URB597 decreased the activity of NADPH and xanthine oxidases in both groups of rats. Moreover, in the heart of SHR rats, URB597 led to an increase of enzymatic and nonenzymatic antioxidant activity and levels (catalase, vitamin C, glutathione/glutathione disulfide [GSH/GSSG]) and upregulation of the thioredoxin system; however, NRf2 expression was downregulated. The opposite effect in relation to Nrf2 activity and the thioredoxin system was observed in DOCA-salt rats after URB597 administration. Despite improvement in antioxidant parameters, URB597 enhanced oxidative modifications of phospholipids (4-hydroxynonenal and isoprostanes) and proteins (carbonyl groups) in SHR heart, whereas 4-hydroxynonenal and carbonyl groups levels decreased in the heart of DOCA-salt rats. Obtained results suggest that examined lipid mediators are involved in peroxisome proliferator-activated receptors (PPAR)-independent and PPAR-dependent modulation of cardiac inflammatory reactions. Furthermore, decreased expression of pro-apoptotic proteins (Bax and caspase 3 and 9) was observed after URB597 administration in the heart of both groups of hypertensive rats, whereas expression of the antiapoptotic protein (Bcl-2) increased in SHR rats. Long-term administration of URB597 altered cardiac redox status depending on the type of hypertension. URB597 enhanced oxidative metabolism and reduced pro-apoptotic factors in the heart of SHR rats, increasing the probability of heart metabolic disorders occurrence or progression.

## 1. Introduction

Hypertension is recognized as the most significant risk factor for cardiovascular diseases. Reactive oxygen species (ROS) play an important role in the redox control of physiological signaling pathways in cardiac cells and the vascular wall. Thus, ROS can also contribute to the development of hypertension [1,2], particularly if ROS activity cannot be adequately countered by cellular antioxidant defense, of which a decrease has been observed in human and in animal models of hypertension [2,3,4]. One of the major cellular processes that is enhanced by high levels of ROS, resulting in dysfunction of myocardial cells, is apoptosis [5,6]. The shift in the redox balance to oxidative conditions leads to oxidative stress in the heart and blood vessels, [3,4] enhancing lipid and protein oxidative modifications that are observed in both human and experimental hypertension [2,3,4]. 

Alterations in phospholipid metabolism, including the formation of ROS-dependent phospholipid peroxidation products and enzymatic metabolism of phospholipid arachidonic acid, leading to the synthesis of endocannabinoids and particularly anandamide, has been observed in hypertension [3,7,8]. Because anandamide levels are regulated by fatty acid amide hydrolase (FAAH)—a major enzyme responsible for the degradation of anandamide [9]—FAAH inhibitors are postulated to be antihypertensive agents [10]. It was previously shown that acute administration of two different FAAH inhibitors, [3-(3-carbamoylphenyl)phenyl] *N*-cyclohexylcarbamate (URB597) [11] and AM35-(4-hydroxyphenyl) pentanesulfonyl fluoride (AM3506) [10] to rats with primary hypertension normalized blood pressure and decreased cardiac contractility. However, chronic administration of URB597 to 11-deoxycorticosterone acetate (DOCA-salt) hypertensive rats reduced blood pressure as well as cardiac and renal hypertrophy in an age-dependent manner but not in SHR rats [12,13]. Endocannabinoids, by activation of cannabinoid receptors, are involved in the regulation of the redox balance and modulation of inflammatory processes [9,14]. Cannabinoid receptor type 1 (CB_1_) activation promotes oxidative stress leading to tissue injury via enhanced inflammation in human cardiomyocytes and coronary artery endothelial cells [15,16]. Nevertheless, cannabinoid receptor type 2 (CB_2_) prevents ROS production and reduces oxidative stress in a mouse model of myocardial ischemia/reperfusion [17]. It was also revealed that chronic administration of URB597 disturbs redox metabolism in the liver of rats with DOCA-salt induced hypertension as well as in the kidney of rats with primary or secondary hypertension [18,19]. 

The primary hypertension animal model corresponds to the hypertension observed in 95% of people with arterial hypertension, whereas the secondary hypertension animal model is strictly related to oxidative and inflammatory stress in the cardiovascular system and corresponds to hypertension associated with other human diseases. Therefore, the aim of this study was to examine the chronic effect of URB597 on local metabolic effects resulting from cardiac redox imbalance in rats with primary and secondary hypertension. Obtained results may allow for better understanding of the molecular processes involved in the development of both types of hypertension. In consequence, targeted pharmacotherapy for both models of hypertension may be suggested. 

## 2. Results

**Figure 1:**
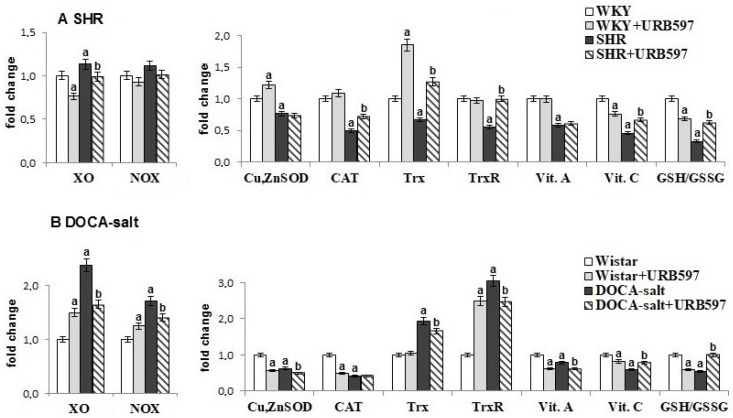
The activity and level of antioxidant parameters in the heart of hypertensive rats (**A**) spontaneous hypertension (SHR) and (**B**) 11-deoxycorticosterone acetate (DOCA)-salt, and rats after administration of [3-(3-carbamoylphenyl)phenyl] *N*-cyclohexylcarbamate (URB597). Data points represent mean ± SD; n = 6; a significantly different from Wistar Kyoto (WKY) rats (*p* < 0.05); b denotes significantly different from SHR (*p* < 0.05); c denotes significantly different from Wistar rats (*p* < 0.05); and d denotes significantly different from DOCA-salt rats (*p* < 0.05).

Metabolic changes observed in hypertension lead to disruption of the redox balance in the heart of rats with spontaneous hypertension (SHRs) and DOCA-salt induced hypertension (Figure 1). 

In the heart of DOCA-salt rats, we observed enhanced activities of enzymes responsible for superoxide anion formation, e.g., cytosolic xanthine and NADPH oxidases. In the heart of SHRs, only xanthine oxidase activity was elevated. These changes were accompanied by alteration of the antioxidant defense system in both groups of hypertensive rats, as a result of decreased activities or levels of antioxidant parameters, such as Cu,Zn-SOD, CAT, vitamins C and A, and the GSH/GSSG ratio. Nevertheless, the response of the thioredoxin system depended on the model of hypertension. The activity of TrxR and the level of Trx were significantly lower in the hearts of SHRs, whereas these parameters observed in the heart of DOCA-salt rats changed in the opposite direction. Administration of URB597 to hypertensive rats decreased the activities of oxidases in the cardiac cells of DOCA-salt rats. Moreover, the activities or levels of most of the examined antioxidants in the heart of SHRs after URB597 administration were increased compared to SHRs. In contrast, the hearts of DOCA-salt hypertensive rats that received URB597 showed a decrease in activities of Cu,Zn-SOD, and TrxR and in the levels of Trx and vitamin A. However, an increased level of vitamin C and GSH/GSSG ratio were observed in this group of animals. 

**Figure 2:**
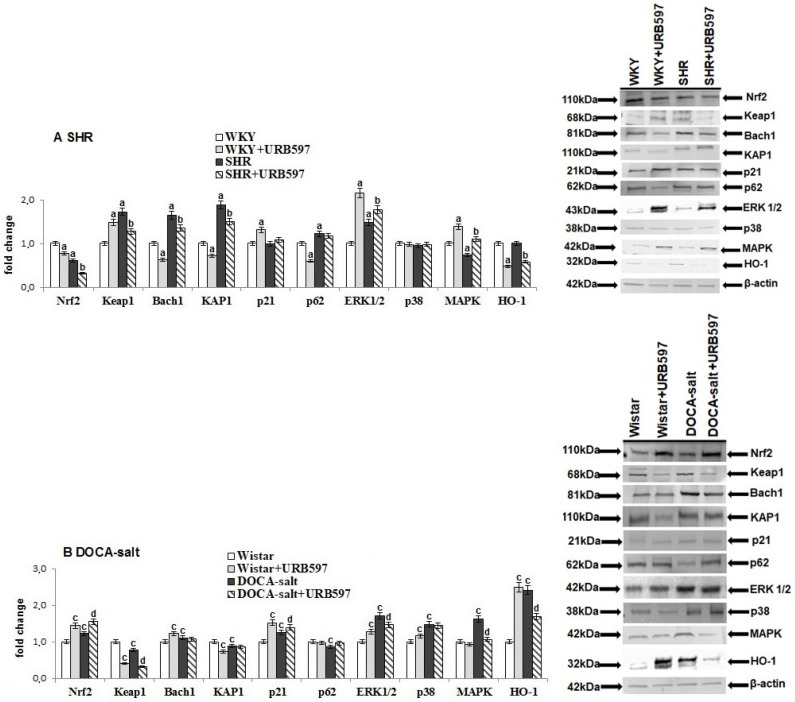
The level of Nrf2 as well as their activators (KAP1, p21, p62, and ERK1/2) and inhibitors (Keap1 and Bach1) and the level of p38, MAPK, and HO-1 in the heart of hypertensive rats (**A**) SHR and (**B**) DOCA-salt, and rats after administration of URB597. The expression of examined protein is shown in comparison to the control.

Independent of antioxidant defense disruption at the molecular level, hypertension also altered the transcription of antioxidant proteins, which is dependent on Nrf2 (Figure 2). This effect was dependent on the hypertension model. An increase in the level of Nrf2 and its target protein, HO-1, was observed in the heart of DOCA-salt rats; whereas in SHRs, the cardiac Nrf2 level decreased. Those changes were associated with disturbances in the expression of Nrf2 inhibitors and activators. Spontaneous hypertension led to an increase in the expression of Nrf2 inhibitors, such as Keap1 and Bach1 in the heart. Furthermore, overexpression of direct activators, such as KAP1, p62, and ERK1/2 was observed in the hearts of SHRs. Similarly, ERK1/2 and p38 expressions were upregulated in the heart of DOCA-salt rats, whereas diverse changes in the expression of Keap1, KAP1, p62 and MAPK were observed in comparison to SHRs. 

URB597 administrated to rats with spontaneous hypertension decreased the expression of cardiac Nrf2, HO-1, Keap1, Bach1, and KAP1; increased expression of kinases ERK1/2 and MAPK; while p21, p62, and p38 amounts were not changed. URB597 given to DOCA-salt rats increased cardiac Nrf2 and p21 expression. Additionally, downregulated expression of Keap1, ERK1/2, and MAPK in the heart of DOCA-salt rats was accompanied by a decrease in the HO-1 level. 

**Figure 3:**
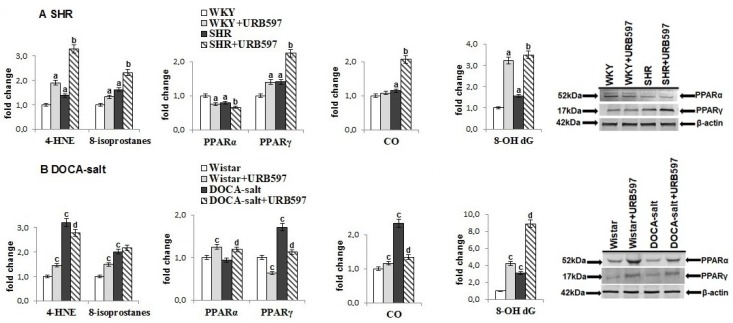
The level of lipid peroxidation products: reactive aldehyde (4-HNE) and prostaglandin derivatives (8-isoPGF_2α_), the level of oxidative modification products of protein (CO-carbonyl groups) and the level of DNA modification (8-OH dG) and expression of PPAR receptors (PPARα and PPARγ) in the heart of hypertensive rats (**A**) SHR and (**B**) DOCA-salt, and rats after administration of URB597. The expression of examined protein is shown in comparison to the control.

The consequence of the observed cardiac redox disturbances was oxidative stress promoting oxidative damage to lipids, proteins, and DNA (Figure 3). In both models of hypertension, a significant increase was observed in the levels of lipid peroxidation products, including compounds generated during oxidative fragmentation (4-HNE) and compounds generated during oxidative cyclization (8-isoPGF_2_). Similarly, oxidative modifications of proteins, estimated as carbonyl groups, as well as DNA guanosine oxidative modifications, measured as 8-OHdG, were also greater than in control groups. Expression of two PPAR forms was different in the heart of rats dependent on the type of hypertension. PPARγ expression was upregulated in both groups of hypertensive rats, whereas expression of PPARα was downregulated in SHRs.

After the administration of URB597 to SHRs and DOCA-salt rats, a further increase in the products of lipid peroxidation and DNA modification in the heart was noted. The only exception was the 4-HNE cardiac level, whose changes were different depending on the hypertension model. In contrast, the level of carbonyl groups in the hearts of DOCA-salt rats decreased after URB597 administration. Moreover, changes in expression of PPARα and PPARγ were diverse in the heart of DOCA-salt hypertensive rats and SHRs receiving URB597.

**Figure 4:**
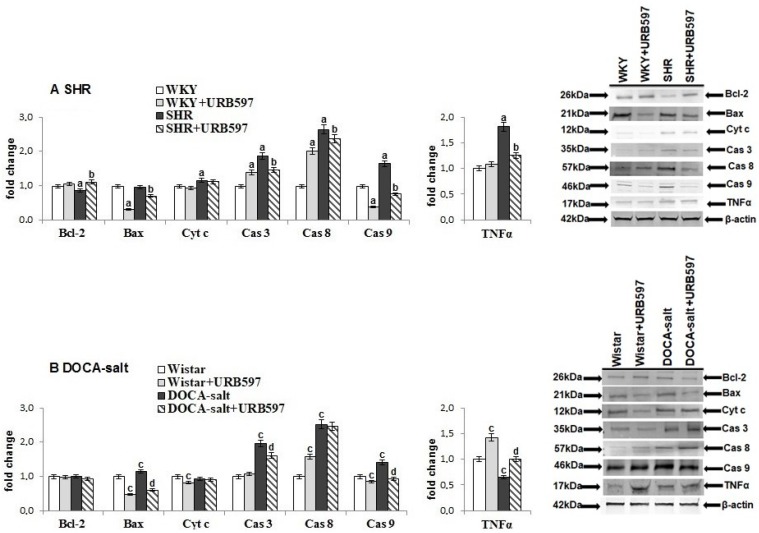
The level of proteins playing essential roles in programmed cell death (Bax, BcL-2, Cyt c, and caspases 3, 8, and 9) and inflammation (TNF-α) in the heart of hypertensive rats (**A**) SHR and (**B**) DOCA-salt, and rats after administration of URB597. The expression of examined protein is shown in comparison to the control.

Consequently, oxidative stress observed in hypertensive rats enhanced pro-apoptotic events (Figure 4). In both groups of hypertensive rats, the level of examined cardiac caspases (3, 8, and 9) was significantly higher than in controls. 

Moreover, in the heart of SHRs, increased expression of cytochrome c and TNF-α and decreased expression of Bcl-2 were observed, whereas in DOCA-salt rats, only Bax expression was higher. URB597 administration decreased the expression of cardiac caspases and of a pro-apoptotic protein (Bax) in the heart of rats in both hypertension models. In contrast, the expression of Bcl-2 was higher only in the heart of rats with primary hypertension. However, changes in the expression of TNF-α were diverse after URB597 administration.

## 3. Discussion

Disturbances in oxidative metabolism are associated with metabolic disorders observed among cardiovascular diseases, including hypertension [2,20]. In the heart of hypertensive rats the tendency for prooxidative conditions is demonstrated by enhanced abilities to form superoxide anions resulting from an increased activity of cytosolic NADPH and xanthine oxidases, particularly in rats with DOCA-salt induced hypertension. Observed changes are in accordance with results of previous studies [21,22]. Moreover, it has been earlier shown that increased activity of NADPH oxidase is involved in the regulation of blood pressure and in turn xanthine oxidase activity participates in heart failure [23,24]. Interestingly, in contrast to obtained results concerning NOX and XO, we also observed increased activity of these major enzymes responsible for ROS in the kidneys of SHR rats in our previous study [19]. However, administration of URB597 resulted in a decrease in the activity of both oxidases in the heart of SHR and DOCA-salt rats. 

Observed upregulation of ROS-generating enzymes was accompanied by disturbances in heart antioxidant defense of the hypertensive rats. Reduced activity of two major antioxidant enzymes (Cu,Zn-SOD and CAT) as well as decreased potency of nonenzymatic antioxidants, such as GSH and vitamins C and A, were observed in the rat hearts in both models of hypertension, as has been shown elsewhere [3,21]. However, URB597 given to hypertensive rats improved antioxidant defense, especially in the heart of SHR rats. We may assume that this effect is associated with enhanced endocannabinoids levels, anandamide in particular, because an increase in the level and activity of antioxidant system parameters has been shown in cells treated with anandamide at different concentrations [25]. Moreover, β-caryophyllene, a naturally occurring CB_2_ receptors agonist, has been shown to prevent the depletion of glutathione and augmentation of antioxidant enzymes: SOD and CAT [26].

Another important cellular redox regulator is the thioredoxin system, consisting of thioredoxin and thioredoxin reductase [27]. Our results reveal that the response of the thioredoxin system depends on the model of hypertension. This system is downregulated in the heart of SHR rats, whereas in DOCA-salt rats, it is upregulated in accordance with previous studies [28]. Such a cardiac cell response may be due to the fact that the increase in the prooxidative enzymes activity in SHR rat hearts was significantly smaller than in the hearts of secondary hypertensive rats. Moreover, the strong positive response of the thioredoxin system to DOCA administration confirms that this cellular system is involved in protection from toxic compounds [29]. URB597 enhanced the thioredoxin system response in SHR heart, but decreased the response in the hearts of DOCA-salt rats. These are the first data indicating that the response of the thioredoxin heart system to hypertension, as well as chronic administration of URB597, in hypertensive rats depends on the type of hypertension.

Despite the above-mentioned effects our study also showed that administration of URB597 to DOCA-salt hypertensive rats promotes enhanced expression of nuclear factor erythroid 2 (Nrf2), the crucial regulator of the basal and inducible expression of antioxidants. However, in the heart of SHRs, the expression of Nrf2 was down regulated. In contrast, our previous study showed that Nrf2 expression in the kidneys of SHR and DOCA-slat rats was not changed [19]. It has been shown that Nrf2 regulates the gene expression of thioredoxin [30]. Thus, observed changes in the thioredoxin system indicate that the DOCA-salt rat heart is devoid of direct antioxidant protection, whereas the SHR rats heart is deprived of transcriptional antioxidant protection. Observed changes in the expression of Nrf2 expression were also related to expression of its inhibitors and activators including kelch-like ECH-associated protein 1 (Keap1), the major cytosolic Nrf2 inhibitor [31]. Reduced expression of Keap1 was observed in the heart of DOCA-salt rats resulting from URB597 administration. However, elevated levels of Keap1 in the heart of SHRs, in combination with decreased expression of mitogen-activated protein kinase (MAPK), leads to diminution of Nrf2 transcriptional activity. The proposed scenario was confirmed in the heart of SHRs by increased expression of basic leucine zipper transcription factor 1 (Bach1), the nuclear inhibitor of Nrf2. URB597 administration reverted observed changes in Keap1 and MAPK expression but Nrf2 expression was not improved. Surprisingly, in comparison to SHR, opposite changes in the expression of Keap1 and MAPK in DOCA-salt rats resulted in increased Nrf2 expression. URB597 improved Nrf2 transcriptional activity causing an additional increase in expression of this nuclear factor. Nrf2 activators, such as KRAB-associated protein-1 (KAP1) and nucleoporin p62 (p62) [32] whose expression in the heart of hypertensive rats is different according to the model of hypertension also regulated Nrf2 transcriptional activity. Nevertheless, upregulation of p38 mitogen-activated protein kinases (p38), MAPK, and extracellular signal-regulated kinases 1/2 (ERK1/2) observed in the heart of DOCA-salt rats, may point to an anti-inflammatory and antioxidant effect, as has been previously demonstrated for several exogenous compounds [33]. 

Evidence from animal studies strongly supports a relationship between redox imbalance and hypertension by increased levels of oxidative stress markers, such as lipid peroxidation products, protein, and DNA oxidative modifications [3,34,35]. An increase in the levels of lipid peroxidation products, such as 4-HNE and 8-isoPGF_2α_, in the heart of hypertensive rats, particularly DOCA-salt rats, was observed in this study. URB597 administration resulted in a greater increase in all oxidative modifications in the hearts of SHR rats, whereas in hearts of DOCA-salt rats, a decrease in 4-HNE was observed. Enhanced oxidative modifications in SHR rat hearts may be connected to mitochondria disturbances by the endocannabinoid system. Anandamide, the concentration which increased after the administration of URB597, by modulating the entry of calcium into the cell, may stimulate the function of the mitochondria, including the production of hydrogen peroxide [36]. In addition, endocannabinoids can stimulate the activity of NOS through cannabinoid receptors and TRPV1 receptors, e.g., in the endothelium [37,38], leading to an increase in superoxide and NO generation. Moreover, fatty acids metabolism by COX and LOX is also associated with enhanced generation of ROS [39,40,41]. In our work on the effect of URB597 on SHR liver metabolism we found an increase in COX2 activity after URB597 [42]. Thus, we may assume that the above-mentioned enzymes may be responsible for ROS generation, which leads to an increase of lipid and DNA oxidation products. At the same time, antioxidant system elements including CAT, Trx/TrxR level/activity and vitamin C and GSH levels, increased. However, these are not the only antioxidants present in the heart. The vitamin A level, which together with vitamin E belongs to the hydrophobic antioxidants, was reduced. Thus, it can be suggested that the protection of membrane phospholipids decreased. In addition, the efficiency of the gene transcription processes antioxidant also decreased (lowering expression of Nrf2 and HO-1), which indicates a decrease in antioxidant activity resulting from the action of antioxidant proteins. Therefore, antioxidant abilities after URB597 administration to SHR may be rather inefficient. 

It is likely that the examined lipid mediators are involved in the cardiac response to URB597 administration via two mechanisms: peroxisome proliferator-activated receptors (PPAR)-independent and PPAR-dependent modulation of cardiac inflammatory reactions. The PPAR-independent mechanism proposed for the hearts of DOCA-salt rats may be associated with a tendency to increase the levels of neuroprostanes which are structural analogs of the anti-inflammatory cyclopentenone prostaglandins. The enhanced formation of reactive aldehydes may inhibit nuclear factor kappa-light-chain-enhancer of activated B cells (NF-κB-mediated pathways [43,44]. Another mechanism is associated with the expression of PPARs [45], as key regulators of inflammation, whose two forms PPARα and PPARγ are diverse in the heart of DOCA-salt hypertensive rats and SHR rats receiving URB597. PPARα shows antioxidant properties by suppressing enzymes involved in ROS/RNS synthesis and/or scavenging what is visible particularly in the hearts of DOCA-salt rats and controls the expression of proteins that participate in inflammatory responses [46]. Therefore, enhanced activation of PPARα observed in the heart of DOCA-salt rats receiving URB597 prevented NF-κB–dependent inflammation. It is likely that this activation may result, in particular, from the action of endocannabinoids, whose levels were elevated after the administration of the FAAH inhibitor, but also the action of some fatty acids and lipid peroxidation products, such as 4-HNE, which may have served as PPARα agonists [46,47]. Moreover, the identification of PPARs in the promoter regions of rats *CAT* and *SOD* genes [48] additionally supports the involvement of these receptors in lowering oxidative stress and lipid peroxidation products. Therefore, the elevated level of PPARα in DOCA-salt rats receiving URB597 may be responsible for reduced cardiac hypertrophy, as observed in our previous study [12,49]. Nevertheless, enhanced activation of the second form, PPARγ, which was observed in the heart of SHR rats receiving URB597 may decrease the observed expression of tumor necrosis factor alfa (TNF-α) as demonstrated earlier [50]. When considering that the function of PPARs is recognized to be important for the development of hypertension, activation of PPARα by URB597 administration in DOCA-salt rats may represent one of the protective mechanisms against the progression of hypertension. 

Apoptosis plays an important role in the pathophysiology of cardiovascular diseases including hypertension [51]. Enhanced apoptotic events were observed in both models of hypertension that were related to myocardial hypertrophy and heart failure [52]. Our study confirmed that oxidative stress in the heart of hypertensive rats also leads to structural modifications of cardiac proteins that may activate apoptosis in SHRs, in particular. The observed increase in TNF-α expression promotes activation of the death receptor-mediated pathway resulting from the significant upregulation of cardiac caspases 8 and 9 in the heart of rats with primary hypertension. Moreover, cardiac cells of rats with primary hypertension were also characterized by downregulation of antiapoptotic protein B-cell lymphoma 2 (Bcl-2) and the release of cytochrome c from mitochondria resulted in caspase 9 activation. These caspases can directly activate executioner caspase 3 [53]. 

Because apoptosis is mediated by components of the endocannabinoid system [9], FAAH inhibitor affected this process. URB597 administered to SHRs diminished heart cell apoptosis by reducing the expression of caspase 8, and consequently caspase 3. Additionally, URB597 reduced the expression of the pro-apoptotic bcl-2-like protein 4 (Bax) and, by increasing the expression of the antiapoptotic Bcl-2 protein, diminished apoptotic events. The expression of caspase 9 was reduced by URB597 administration in both groups of hypertensive rats. Conversely, it has been reported that, in pathological conditions, the apoptotic cell death markers (caspases 3 and 7 activity and DNA fragmentation) in FAAH knockout mice were increased, but these effects were attenuated by CB_1_ receptor antagonists [9]. Nevertheless, analysis of caspase expression changes in the heart of rats with hypertension indicates that URB597 administration to SHR rats prevented apoptotic events involved in both extrinsic and intrinsic cell death mechanisms, whereas in DOCA-salt rats, URB597 mainly influenced the intrinsic death pathway. 

## 4. Materials and Methods

### 4.1. Materials

Drugs and reagents were obtained from the following sources: 3-(3-carbamoylphenyl)phenyl *N*-cyclohexylcarbamate (URB597), 4-hyroxynonenal (4-HNE), 8-iso Prostaglandin F_2α_-d_4_ (8-isoPGF2α–d_4_), 8-iso Prostaglandin F_2α_ (8-isoPGF2α) and 8-hydroxy-2′-deoxyguanosine (8-OHdG) from Cayman Chemical Company (Ann Arbor, MI, USA); 11-deoxycorticosterone acetate (DOCA), dimethyl sulfoxide (DMSO), *N*,*N*-dimethylformamide (DMF), β-Nicotinamide adenine dinucleotide 2′-phosphate reduced tetrasodium salt hydrate (NADPH), xanthine, xanthine oxidase (XO), 9,9′-Bis(*N*-methylacridinium nitrate) (lucigenin), superoxide dismutase (SOD), catalase (CAT) thioredoxin (Trx), l-ascrobic acid, retinol, l-glutathione reduced (GSH), l-glutathione oxidized (GSSG), benzaldehyde-d_6_ and Tween 80 were acquired from Sigma-Aldrich (Steinheim, Germany); pentobarbital sodium was purchased from Biowet (Puławy, Poland); chloro-2,4-dinitro benzene (CDNB), butylated hydroxytoluene (BHT), and 5,5′-dithiobis (2-dinitrobenzoic acid) (DTNB) were acquired from Sigma-Aldrich (Steinheim, Germany). DOCA was dissolved in DMF, whereas URB597 was dissolved in an URB597 solvent: a mixture of DMSO, Tween 80, and saline (0.9% NaCl) [1:2:7; *v*/*v*/*v*]. 

#### 4.1.1. Animals

The experiment was performed on age-matched male rats with comparable primary hypertension (SHRs) and rats with secondary (DOCA-salt) hypertension in order to distinguish changes induced by hypertension from those related to any one particular hypertension model. The DOCA-salt model was chosen because in this model of hypertension, cardiovascular remodeling characteristic of human volume-overload is observed. Rats were housed with free access to standard pelleted rat chow and water and were maintained in a 12/12 h light/dark cycle. All procedures and experimental protocols were approved by the local Animal Ethics Committee in Białystok, Poland (resolution No. 4/2012 of 25.01.2012).

#### 4.1.2. SHRs Group

Experiments were performed on 8- to 10-week-old male SHRs (270–350 g) and normotensive control male (290–390 g) Wistar Kyoto rats (WKY). 

The rats were randomly subdivided into four groups each consisting of six rats as follows:

1A: WKY rats treated intraperitoneally (i.p.) with the URB597 solvent (vehicle) every 12 h for the last 2 weeks of the study period,
2A: WKY rats treated i.p. with URB597 every 12 h for the last 2 weeks,
3A: SHRs treated i.p. with the URB597 solvent (vehicle) every 12 h for the last 2 weeks, and
4A: SHRs treated i.p. with URB597 every 12 h for the last 2 weeks.

#### 4.1.3. DOCA-Salt Group (DOCA-Salt Hypertensive Rats)

Young male Wistar rats aged 4–5 weeks, initially weighing 100–140 g, were used in this experiment. The rats were anesthetized by an intraperitoneal (i.p.) injection of pentobarbital sodium (70 mg/kg body weight; b.w.) and unilaterally nephrectomized. After a 1-week recovery period, the animals were given twice weekly a subcutaneous (s.c.) injection of DOCA (25 mg/kg b.w. in 0.4 mL DMF/kg b.w.) for 6 weeks and given salt in the form of a 1% NaCl solution as a substitute for drinking water to induce hypertension. Control rats (normotensive Wistar rats) received s.c. injection of the vehicle for DOCA (0.4 mL DMF/kg b.w.) twice weekly for 6 weeks and drank tap water. Four weeks later, one group of DOCA-salt rats and one group of Wistar rats were injected i.p. every 12 h with URB597 (1 mg/kg b.w. in 1 mL of the URB597 solvent/kg b.w.) for 2 weeks [54,55].

The rats were randomly subdivided into four groups each consisting of six rats as follows:

1B: normotensive, uninephrectomized control Wistar rats (DMF given s.c. for six weeks and URB597 solvent for the last 2 weeks of the study period);
2B: normotensive rats that received s.c. DMF for 6 weeks and URB597 (i.p.) for last 2 weeks,
3B: DOCA-salt hypertensive rats (DOCA-salt given s.c. for 6 weeks); and
4B: DOCA-salt hypertensive rats treated i.p. with URB597 for the last 2 weeks.

### 4.2. Determination of Blood Pressure in Conscious Rats 

Systolic blood pressure (SBP) was determined in conscious rats via the noninvasive tail-cuff method using the Rat Tail Blood Pressure Monitor from Hugo Sachs Elektronik-Harvard Apparatus (March–Hugstetten, Germany) before the first dose of URB597 or its vehicle and 12 h after the final dose. Two weeks of URB597 administration did not modify SBP in SHR and WKY but it tended to reduce SBP in DOCA-salt hypertensive rats and did not affect SBP in Wistar rats [12,13].

### 4.3. Confirmation of URB597 Action (Anandamide Level Determination)

To confirm the effect of FAAH inhibition by URB597, the anandamide level was determined by liquid chromatography-mass spectrometry (LC-MS) in both models of hypertension. The levels of anadamide in the heart of rats with spontaneous hypertension were as follows: WKY, 92.3 ± 7.7 fmol/mg tissue; WKY + URB597, 110.0 ± 6.0 fmol/mg tissue; SHR, 71.6 ± 10.6 fmol/mg tissue; and SHR + URB597, 137.0 ± 7.9 fmol/mg tissue. However, cardiac anandamide levels of DOCA-salt rats were as follows: Wistar, 60.2 ± 1.2 fmol/mg tissue; Wistar+URB597, 104.1 ± 15.9 fmol/mg tissue; DOCA-salt, 99.0 ± 0.7 fmol/mg tissue; and DOCA-salt+URB597, 111.1 ± 3.5 fmol/mg tissue.

### 4.4. Tissue Preparation for Biochemical Examinations

Twelve hours after the final dose of URB597 (or its vehicle), the rats were anesthetized with an i.p. injection of pentobarbital (70 mg/kg b.w.) and euthanized to collect tissues samples. The hearts were perfused with 0.9% NaCl and were cut in half longitudinally into two equal size and quality halves. The first part of the fresh tissue was snap-frozen, pulverized and held at −80 °C. The other part was homogenized in saline and next centrifuged at 20,000× *g* for 15 min at 4 °C. 

### 4.5. Determination of Pro-Oxidant Protein Activity 

NADPH oxidase (NOX EC.1.6.3.1) activity in the cardiac homogenates was measured by detecting the production of the superoxide anion by a lucigenin-derived chemiluminescence assay with NADPH as a substrate. Superoxide production is expressed in relative light units per milligram of protein (RLU/mg) [56].

Xanthine oxidase (XO EC.1.17.3.2) activity was measured as the rate of uric acid production when xanthine was incubated with tissue homogenates. One unit of XO activity was defined as 1 µmol of uric acid produced per minute at 37 °C according to absorbance at 292 nm [57].

### 4.6. Determination of Antioxidant Protein Activity/Level 

Superoxide dismutase (Cu/Zn–SOD EC.1.15.1.1) activity was assayed according a method published elsewhere [58]. The oxidation of epinephrine was performed in terms of the production of adrenochrome, which has an absorption maximum at 480 nm. One unit of SOD is defined as the amount of the enzyme that inhibits the rate of autoxidation of epinephrine by 50%.

Catalase (CAT EC.1.11.1.9) activity was measured in cardiac homogenates by spectrophotometric analysis (at 240 nm) of the rate of hydrogen peroxide decomposition, according to a method published previously [59]. One unit of CAT is defined as the amount of the enzyme necessary to catalyze the decomposition of 1 µmol of hydrogen peroxide to water and oxygen within 1 min. 

Thioredoxin (Trx) was quantified by ELISA method [60]. Spectral absorption was read at 450 nm with the reference filter set to 620 nm. 

Thioredoxin reductase (TrxR EC.1.8.1.9) activity was measured by means of a commercial assay kit (Sigma-Aldrich, St. Louis, MO, USA) that colorimetrically estimates the reduction of 5,5′-dithiobis(2-nitrobenzoic) acid by NADPH to 5-thio-2-nitrobenzoic acid [61]. 

### 4.7. Detection of Protein Antioxidants Levels

High performance liquid chromatography (HPLC) with ultraviolet (UV) detection was applied to quantify vitamin C [62] and vitamin A [63]. UV detection was performed at 250 nm for vitamin C and at 298 nm for vitamin A.

The levels of reduced and oxidative glutathione (GSH and GSSG, respectively) were measured according to the procedure of Maeso using capillary electrophoresis (CE). The separations were performed on a fused silica capillary with an ultraviolet detector set at 200 nm [64].

### 4.8. Determination of Lipid, Protein, and DNA Modifications

Lipid peroxidation was assessed by gas chromatography with the tandem mass spectrometry (GC-MS/MS) method published previously [65], with a modification. The method is based on the use of *O*-(2,3,4,5,6-pentafluorobenzyl) hydroxylamine hydrochloride (PFBHA⋅HCl) to form the *O*-pentafluorobenzyl-oxime (PFB-oxime) derivative of 4-hydroxy-nonenal (4-HNE). Quantitation was implemented using benzaldehyde-D_6_ as an internal standard in selected ion-monitoring (SIM) mode. The ions were 333.0 and 181.0 *m*/*z* for 4-HNE-PFB-TMS and 307.0 *m*/*z* for IS (benzaldehyde-D_6_) derivatives.

The total level of F_2_-isoprostanes (8-isoPGF_2α_) was estimated by a liquid chromatography with mass spectrometry (LC-MS) [66]. Due to the presence of a carboxylic group, electrospray ionization (ESI) operating in negative ion mode with multiple-reaction monitoring (MRM) mode provided the best sensitivity. For MRM analysis, the mass transitions 353.2→193.1 *m*/*z* for 8-isoPGF_2__α_ and 357.2→197.1 *m*/*z* for 8-isoPGF_2__α_-d_4_ were selected. 

Protein oxidative modifications (carbonyl groups) were determined according to the method published previously [67]. Carbonyl content was calculated from peak absorption (370 nm) using 2,4-dinitrophenylhydrazine as a reagent.

8-hydroxy-2′-deoxyguanosine (8-OHdG) was quantified by LC-MS [68], with a modification. Genomic DNA was isolated from tissue samples using a regular commercial DNA extraction kit and a standard protocol (Sigma’s GenElute Mammalian Genomic DNA Miniprep Kit, St. Louis, MO, USA). DNA concentration was measured by UV spectroscopy. 8-OHdG was analyzed in positive-ion mode with MRM. Transitions of the precursor to the product ion were as follows: 284.1→168 *m*/*z* (quantifier ion) and 284.1→69 *m*/*z* (qualifier ion).

### 4.9. Western Blot Analysis

Cellular proteins (Nrf2, Keap1, Bach1, KAP1, p21, p62, ERK1/2, p38, MAPK, HO-1, Bcl-2, Bax, cytochrome c, PPARα, PPARγ and caspases 3, 8, and 9) were extracted from myocardial tissue homogenates and analyzed according to a method published previously [69]. The expression of all proteins was analyzed in the cytosolic fraction. Samples containing 30 µg of total protein were mixed with sample loading buffer (Laemmli buffer containing 5% β-mercaptoethanol), heated at 95 °C for 10 min, and separated by sodium dodecyl sulfate–polyacrylamide gel electrophoresis (SDS-PAGE) in a 10% gel with Tris-glycine buffer. The same procedure was carried out to prepare the negative control (containing pure phosphate buffered solution) and the positive control (purchased complete cell lysate; Santa Cruz Biotechnology, Santa Cruz, CA, USA). As internal loading controls, β-actin was used. After the separation, the proteins were electrophoretically transferred onto nitrocellulose membranes. The blotted membranes were blocked with 5% skim milk in TBS-T buffer (Tris-buffered saline with 0.05% Tween 20) for 1 h and cut into fragments corresponding to the selected molar masses of the proteins. Primary antibodies against MAPK1 (WH0005594M1, host: mouse, 1:1000), Bcl-2 (B3170, host: mouse, 1:1000), HO-1 (H4535, host: mouse, 1:200), Bach1 (A08321, host: rabbit, 1:500), KAP1 (SAB4502351, host: rabbit, 1:1000), PPARα (SAB4502260, host: rabbit, 1:400) and β-actin (A2228, host: mouse, 1:1000)were purchased from Sigma-Aldrich (St. Louis, MO, USA). Primary antibodies against Keap1 (SC-15246, host: goat, 1:1000), Bax (SC-526, host: mouse, 1:1000), and cytochrome c (SC-13561, host: mouse, 1:1000), were purchased from Santa Cruz Biotechnology, (Santa Cruz, CA, USA) and primary antibodies against caspase 3 (AF-605-NA, host: goat, 1:2000) and Nrf2 (MAB3925, host: mouse, 1:500) were purchased from R&D System (Minneapolis, MN, USA). Primary antibodies against ERK1/2 (9106, host: mouse, 1:1000) were purchased from Cell Signaling (Danvers, MA, USA); against p38 (bs-0637R, host: rabbit, 1:1000) and caspase 9 (ABIN1532234, host: rabbit, 1:1000) from Antibodies-online.com (Atlanta, GA); against caspase 8 (NBP1-05123, host: rabbit, 1:2000) from Novus Bio (Littleton, CO, USA); against p21 (ab109199, host: rabbit, 1:1000) and PPARγ (ab59256, host: rabbit, 1:400) from Abcam (Cambridge, MA, USA); against p62 (orb89844, host: rabbit, 1:1000) from BiorByt (Cambridge, UK). Secondary antibodies against rabbit (A3687, host: goat, 1:2000) or mouse (A3562, host: goat, 1:2000) were purchased from Sigma-Aldrich (St. Louis, MO, USA). Visualized protein bands were quantitated on a Versa Doc System with Quantity One software (Bio-Rad Laboratories Inc., Hercules, CA, USA). The results are expressed as a percentage of the expression determined in control groups. 

### 4.10. Statistical Analysis

The data are expressed as mean ± SD. Statistical comparisons were performed by two-way analysis of variance (ANOVA) followed by the post hoc Tukey test. The results were considered statistically significant if the *p* values were 0.05 or less.

## 5. Conclusions

In summary, these studies indicate that chronic administration of FAAH inhibitor, URB597, to hypertensive rats modifies the cardiac redox balance depending on the type of hypertension. URB597 enhanced oxidative status and reduced pro-apoptotic parameters in the hearts of rats with primary hypertension, but partially prevented oxidative stress in the hearts of rats with secondary hypertension. This was accompanied by an immune response promoted by PPARs dependent on oxidative alterations in each type of hypertension. Therefore, long-term administration of URB597 led to an increased probability of heart metabolic disorders occurrence or progression, especially in the case of spontaneous hypertension, which is the most common type of hypertension in humans.
